# Steric exclusion and constraint satisfaction in multi-scale coarse-grained simulations

**DOI:** 10.1016/j.compbiolchem.2016.06.007

**Published:** 2016-10

**Authors:** William R. Taylor

**Affiliations:** Francis Crick Institute, 1 Midland Rd., London NW1 1AT, UK

**Keywords:** Coarse-grained molecular modelling, Steric-exclusion, Constraint satisfaction

## Abstract

An algorithm is described for the interaction of a hierarchy of objects that seeks to circumvent a fundamental problem in coarse-grained modelling which is the loss of fine detail when components become bundled together. A “currants-in-jelly” model is developed that provides a flexible approach in which the contribution of the soft high-level objects (jelly-like) are employed to protect the underlying atomic structure (currants), while still allowing them to interact. Idealised chains were used to establish the parameters to achieve this degree of interaction over a hierarchy spanning four levels and in a more realistic example, the distortion experienced by a protein domain structure during collision was measured and the parameters refined. This model of steric repulsion was then combined with sets of predicted distance constraints, derived from correlated mutation analysis. Firstly, an integral trans-membrane protein was modelled in which the packing of the seven helices was refined but without topological rearrangement. Secondly, an RNA structure was ‘folded’ under the predicted constraints, starting only from its 2-dimensional secondary structure prediction.

## Highlights

• The method developed here provides a fast and flexible way to capture the structure of most macromolecules in a hierarchy of increasingly larger coarse-grained levels without losing the detailed low-level representation.
• Molecules can then be viewed using an integral graphical viewer or animated through a high-level application programming interface (API) in C++.
• Although much testing remains to be done, the system has the potential to be applied to very large dynamic systems including both protein and nucleic acids.

## Graphical abstract

**Figure:**
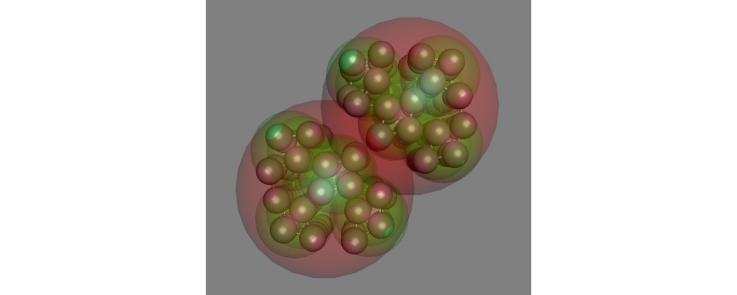

## 1 Overview and model design

### 1.1 Overview

#### 1.1.1 Introduction

Molecular simulation methods are increasingly being applied to ever larger systems. However, given finite computational resources, there is inevitably a limit to the size of the system that can be simulated in a reasonable time. Besides buying bigger computers, approaches to circumvent this limitation generally follow the original approach of Michael Levitt and Warshel (1975) and reduce the number of simulated particles by combining groups of atoms, such as an amino acid side-chain, into a single pseudo-atom with a spherical radius that reflects the volume of the combined atoms (Bond et al., 2007, Izvekov and Voth, 2005) (see Ref. Tozzini, 2005 for a review). This coarse-grained approach suffers from the problem that as the number of combined atoms increases in number, so their representation becomes less realistic. Taken to an extreme degree, if a protein is represented by a single sphere, then all the details of its structure become hidden.

In this work, I describe the development of an earlier algorithm for collision detection between groups of multiple points (Katsimitsoulia and Taylor, 2010, Taylor and Katsimitsoulia, 2010) into a general method that allows points (atoms^1^) to be contained within a variety of shapes but still retaining the property that the interaction (or collision) of these higher-level objects is based on contact between their component atoms. The approach follows a divide-and-conquer strategy in which the problem of dealing with a quadratic computational complexity in the number of points is reduced by partitioning the interactions into a series of grouped interactions on a hierarchic tree.

#### 1.1.2 Hierarchic collision detection

Fast collision detection in interactive computer game simulations is enabled by the use of a bounding-box construct (Teschner et al., 2005). This is a box in which a group of points is contained and the calculation of the interactions between points in different boxes is not evaluated until their boxes overlap. This approach is similar to, but distinct from, the use of neighbour-lists in molecular dynamics (MD) (Verlet, 1967). In general, the bounding box can be any shape but should, ideally have a simple shape to allow for fast overlap calculation. The advantage of a box that is aligned with the coordinate frame is that only *X*, *Y*, *Z* values need be compared, without the more costly calculation of a 3D distance. Unfortunately, unlike objects in computer games, in the molecular world the dominant orientation dictated by gravity is absent so a construct based on the “world” coordinate frame is less relevant (see Ref. Muth et al., 2007 for a review).

Previously, this approach was used to speed collision detection between atoms using a simple spherical “box” (Taylor and Katsimitsoulia, 2010) but when dealing with non-spherical objects, such as alpha-helices of nucleic acid segments, a sphere is not an ideal shape and when made large enough to enclose an elongated object many other objects can be brought into the calculation even when they are far from interacting, especially if they too are elongated. In this work, the original method based on spheres is extended to a wider variety of shapes and into a generalised hierarchy in which the boxes themselves can be assigned different collision properties at any level in the hierarchy.

For example; attributing a box object with hard-shell collision behaviour is equivalent to ignoring all their internal components when two objects collide. If this absolute degree of repulsion is softened, then the objects can now partly interpenetrate, allowing their internal components to come into contact, with their own collision properties contributing to the interaction. If the high-level objects do not repel at all, then all repulsion will be determined by the structure and properties of the internal components.

Through this approach, the coarse-grained representation at the high-level does not completely mask the details of the interaction at the lower level, however, it is still being used to save computation time, especially where it retains a good shape-match to the interacting surface of its components.

### 1.2 Model specification

#### 1.2.1 Object descriptions

The choice of shapes for the higher level objects (or containers) is, in principle, not limited but simple shapes have been chosen that reflect those encountered in biomolecular models. These include a sphere, which can be generalised as an oblate or prolate ellipsoid^2^ (but not scalene, as will be discussed below) and a tube (or more precisely, a fixed-length straight section of pipe with hemi-spherical end-caps). Both spheres and tubes have been used widely in coarse-grained modelling, with the latter being a good representation for an RNA stem-loop (Ding et al., 2008) or an *α*-helix (Minary and Levitt, 2010) or even a general peptide (Vácha et al., 2014).

Objects at any level can adopt any mix of these basic shapes which can be different sizes and for ellipsoids, have different degrees of eccentricity (from “cigar” to “flying saucer”), similarly, tubes can have differing length:radius ratios (from “coin” to “pencil”). However, as tubes have hemispherical end-caps, they approach a spherical shape as their length decreases, as do ellipsoids as their axes become equal. For each of these objects and their pairwise interactions, it is necessary to have a fast algorithm to compute their surface and the point at which their surfaces make contact. For spheres, both these are trivial and between fixed-length straight tubes, both with themselves and spheres, the calculations require only slightly more “book-keeping”. However, the interaction of ellipsoids is less simple and an analytic solution for the contact-normal between two ellipsoids is not trivial (Donev et al., 2004, Kallrath, 2015).

#### 1.2.2 Coupling the levels

It would be of little use if objects were to wander outside their container as their interactions would not be detected until their containers eventually made contact. Although this might be avoided or reduced by having a large container, for computational efficiency, it is better if the container maintains a close fit to its contents.

The task of ensuring a good match between the parent container and its enclosed children was obtained it two main ways: firstly, by maintaining the centre of the parent container at the centroid of its children and secondly, by applying any displacement or rotation of the parent automatically to all its children and recursively to any children that they also contain. However, while these two conditions serve to synchronise the motion between levels, they do not prevent the escape of children through isotropic diffusion. This was tackled directly by applying a corrective push to return any wayward children back into the parental fold. With a few minor elaborations described below, these couplings constitute the only direct connection between levels in the hierarchy of objects as no collisions are calculated between objects at different levels.

Despite their simplicity, these couplings are sufficient to generate appropriate behaviour during collisions. For example; if at one extreme, the parent shapes interact as hard surfaces then in a collision, all their contents will move with them during recoil. At the other extreme, if the parents have no repulsion but their children do, then the parent containers will interpenetrate, allowing collisions to occur between the children which will repel each other and as a result, their centroids will separate, with the centres of their parents tracking this displacement. In the intermediate situation, where the parents retain some repulsive behaviour, their inter-penetration will be reduced like the collision of two soft bodies but retaining the hard-shell repulsion between children.

An option was added to exaggerate this “currants-in-jelly” collision model by making the soft parental repulsion dependent on the number of colliding children. This allows two parental containers to pass through each other undeflected until their children clash: at which point the parental repulsion becomes active and guides the two families of points apart before extensive interaction occurs between the children. Such behaviour prevents the separation of the parents being completely dependent on the displacement of the children as in high speed^3^ collisions, any internal structure within the children can be disrupted before separation is attained.

A further safeguard was introduced to deal with the possible situation where two families of points have collided and overlapped to such an extent that there is no dominant direction of separation provided by the children. To avoid this family grid-lock, children with different parents were not repelled along their contact normal but each was given an additional vectorial component back towards their parental centre.

#### 1.2.3 Parental realignment

The way in which parents track their children was described above only in terms of translation, but for objects other that the sphere, a rotational realignment must also be considered. Any parental rotation is automatically communicated to its children but a rotation of the children, either caused randomly or by a push or pull on a group of bonded children, does not get communicated up to the parent in the same way.

However, for both the ellipsoid and tube, there is a unique axis of symmetry and this can be recalculated from the configuration of the children. If the children are bonded in a chain then the axis can be reset simply by considering a small group of positions around the termini (or jointly the complementary 5′, 3′ pairs in double-stranded nucleic acids). More generally, the axis can be recalculated from the moments of inertia of the point set, irrespective of their connectivity. This computationally more expensive calculation was made much less frequently compared to the positional tracking up-date.

### 1.3 Shape correspondence to molecular objects

Although the mapping of the objects described above to molecular substructures is quite arbitrary and could be devised “from scratch” with each application, there are some natural associations and in the program that implements the methods (called **SimGen**), the construction of these have been facilitated by specialised routines that parse the input stream. (See Taylor and Katsimitsoulia, 2012 for an outline).

#### 1.3.1 Proteins

The “atomic” level for protein structure is assumed to be the chain constructed on consecutive *α*-carbon positions. By default, the atomic level is always a hard-sphere and in the internal coordinate representation of 0.6 units to 3.8 Å(the *α*-carbon–*α*-carbon ‘bond’ distance), the bump-radius is set to 3 which is the closest approach distance between positions *i* and *i* + 2. Bonded positions (*i*, *i* + 1) do not bump.

The natural object to represent secondary structure elements is a tube. Preset length:radius ratios are adopted for alpha, beta and coil structures with the ratio determined by the axial displacement per residue for each secondary structure which is 1.5 and 3.0 for alpha and beta, respectively. Given the more irregular nature of the coil regions, an arbitrary value of 0.5 was used but the coil residues were only weakly constrained to keep within this (short) tube.

Domain level structure is best represented by an ellipsoid (Taylor et al., 1983). Although the average domain shape is a scalene ellipsoid (semi-axes *A* ≠ *B* ≠ *C*) (Aszódi and Taylor, 1994), the program picks the closest symmetric prolate ellipsoid (*A* ≠ *B* = *C*) or oblate ellipsoid (*A* = *B* ≠ *C*).

The overall protein envelope (enclosing multiple domains, when present) is again generally ellipsoidal, but this and higher level (quaternary) assemblies are best determined on an individual basis.

#### 1.3.2 Nucleic acids

The atomic level representation for DNA and RNA was taken as the phosphate atom and in the internal scale, the *P*–*P* distance is 1 unit.

In double stranded RNA and DNA it is desirable to have base-paired phosphates move as a linked entity and this was achieved by enclosing them at either end of a tube. As well as linking the phosphates, the tube provides a volume to mimic the bulk of the (unrepresented) nucleotide bases. The bulk of the bases should really lie between the midpoints of the *P*–*P* virtual bonds and in the refinement the double helix geometry, which are distinct in RNA and DNA, this difference was accommodated in the choice of ideal distances and angles.

Segments of base-paired phosphate ladders are again best represented as a tube which is a good model for the hairpins (stem-loops) found in RNA but to represent an extended chain of DNA, a succession of segments (like a string of sausages) was used that allowed the double-helix of the phosphate backbone to progress uninterrupted from one to the next.

## 2 Algorithms and implementation

### 2.1 Collision algorithms

In the **SimGen** program, the **bumper** routine has the task of identifying objects that have approached closer than permitted and repelling them by a fixed-size “kick” specified in the parameter file. It must overcome two difficulties: firstly, for large numbers of objects, it is computationally too expensive to compare all-against-all and, secondly, the objects do not all have simple shapes so the surface–surface distance between all combinations of object shapes must be accommodated.

#### 2.1.1 Avoiding *N* × *N*

The **bumper** routine uses the hierarchic structure of the data to avoid a *N*^2^ order calculation between all pairs of objects. Beginning at the highest level (1) all objects at that level are compared pairwise but only if two objects at this level are in collision, are their children then considered. As discussed in the Introduction, in computer-graphics terms, the high-level objects are the bounding-volumes for their children.

However, before the parents are repelled, a calculation is made of how many collisions there are between their children. These two distinct calculations of collisions within a family and collisions between families are performed by bumpin() and bumpex(), respectively.

**bumpin()** :

If the number of children in a family is less than 20, bumpin() uses a simple pairwise algorithm (in getBumps()) to provide a list of objects that are potentially in collision. With more children, then an approximate algorithm is used that is based on the partially sorted *X*, *Y*, *Z* lists maintained internally in **SimGen**. (See source code). This selection is based on the largest dimension of the object: the maximum axis length for an ellipsoid or the larger of the length and diameter of a tube. The list is sorted by degree of violation so bumpin() will deal with the worst cases first.

bumpin() firstly checks the true separation of the two objects (a,b) using touch() which returns a negative distance if the objects inter-penetrate. (See further sections below for details of each type of interaction). If a and b are not atoms, then a count (m) is made of how many collisions occur between their children using bumpex(). Two parameters, *hard* and *soft*, are specified in the input that set the repulsion step for each type of object. If both parameters have been given values, then the degree of repulsion is calculated based on the number of colliding children, m, as: boot = f * soft + (1 − f) * hard; where f = exp(−m * m/100);. The resulting value of boot is then used by the utility part2cells() to push the objects symmetrically back towards a distance (d) where they are no longer in collision. (See Box 1 for pseudo code.^4^)

Both bumpin() and getBumps() employ a filter encoded in exempt() that is TRUE if the two objects are exempt from collisions, for example, if they are bonded or linked. In bumpin(), however, exempt() is only called after bumpex() as the children (and their offspring) inside two exempt objects might well be in collision.

**bumpex()** :

The bumpex() routine evaluates each pair of children between their two colliding parents and returns the number of collisions. As it does so, it also takes steps to rectify the situation by separating the clashing children. As it is known to which parent each child belongs, they are also given a nudge back towards their parent before being separated. The strength of these kicks depend on both the *hard* and *soft* parameter values: the nudge back home is always *soft*/10 while the separation is *hard* at the atomic level and *soft* for higher levels (See Box 2).

Once bumpin() has completed at one level, it is called recursively and continues to traverse the hierarchic tree of objects in a depth-first order. The application of the bumpin()/bumpex() pair of routines is not recursive: i.e.: bumpex() does not re-call bumpin() on colliding children. However, bumpex() is itself recursive and is called on the children of any clashing children that it encounters.

#### 2.1.2 Calculating contacts

**SimGen** employees three object types, giving six possible types of encounter which are dealt with by the touch() routine (Box 3).

*Sphere/sphere:*

A pair of spheres are the simplest case, with surface contact made at the average distance of their bumping diameters. (Or the sum of the corresponding radii: *R*_*ab*_ = *R*_*a*_ + *R*_*b*_, for two objects *a* and *b*).

*Sphere/tube:*

For a sphere and a tube, the closest approach is the shortest sphere-centre to tube axis line-segment, less their joint radii (*R*_*ab*_). If a perpendicular construction from the sphere centre to the axis line lies within the tube end-points then this, less *R*_*ab*_, is the closest approach of their surfaces. Otherwise, it is simply the shorter tube-end to sphere distance (less *R*_*ab*_).

*Sphere/ellipsoid:*

The distance of a point from the surface of a general (scalene) ellipsoid in not trivial, however, as **SimGen** only deals with spheroids, a simple construction based on the foci of their ellipse-of-rotation can be used to decide if a point is inside or outside the ellipsoid. A path from one focus to any point on the ellipse and back to the other focus, has a constant length. (A property often exploited to draw an ellipse with a fixed length of string.) The distance of the foci from the centre can be solved from the lengths of the axes, *A* and *B*, as: *c* = √ (*a*^2^ − *b*^2^), where *a* and *b* are the semi-axis lengths. (see construction and details in Box 4). So if the summed distance from any point to the foci is longer than the ‘string’ length, it is outside and, if less, it is inside.

The sum of the foci distances less the ‘string’ length is zero on the surface but elsewhere is not the true distance to the surface. However, when scaled by 1.4, this value is a good approximation to the true distance to the surface for both prolate and oblate ellipsoids and is the value returned by the routine inEgg(), which encodes this algorithm. In its most minimal form, the algorithm only needs to calculate two distances but as the foci positions are not stored these are also calculated.

In the range 0, …, *R*_*ab*_ a more complicated but accurate routine vec_to_egg() is used to return the true value of the distance between the surfaces.

*Tube/tube:*

If a mutual perpendicular line (the contact normal) is constructed between the axes of a pair of tubes and if the ends of this lie between the end-points of the tubes, then this is the closest approach. Otherwise one of the four end-end distances will be shortest. The shortest distance, less *R*_*ab*_ is the distance between the surfaces.

*Tube/ellipsoid:*

The distance of a tube to an ellipsoid is found using the same algorithm described above for a point (sphere) and ellipsoid (inEgg()) by iteratively bisecting the line between the tube end-points.

Starting with the three end–mid–end points along the axis (*p*1, *p*2, *p*3), then if any corresponding distance (*d*1, *d*2, *d*3) returned by inEgg() is negative, there is a clash. Otherwise, the point associated with the largest value can be excluded and the calculation repeated with the remaining two points and their midpoint (Box 5). The algorithm converges rapidly and is stopped when the points get too close. At the end, the true distance to the ellipsoid surface is returned using the more complicated vec_to_egg() routine (since the inEgg() value is only exact at the surface).

*Ellipsoid/ellipsoid:*

Surprisingly, there is no analytic solution for the contact normal between two ellipsoid surfaces as the expression for this is a quartic equation that requires a numerical solution. A solution probably could be found for the more symmetric case of two spheroids but instead, a fast iterative algorithm was used to find the contact normal using a recursive division approach that is an extension of that used for the simpler tube/ellipsoid problem.

Rather than iteratively bisect a line, as was done on the tube axis, extending the approach to a surface leads to the iterative subsection of a triangle – or rather two, as there are two ellipsoids to consider. If the triangles are trisected using a mid-point/vertex construction, the sub-triangles become progressively elongated. To avoid this, an internal triangle was constructed from the mid-points of each edge – so strictly, each triangle is quad-selected (see Box 6).

A starting set of triangles was obtained from the end-points of the axes, giving 8 triangles per ellipsoid and a starting pair was selected which had the shortest mid/mid point distance. In all the iterations, the mid point is not simply the mean of the vertices but is the point where the extension of a line from the centre through this point cuts the ellipsoid surface. The utility routine sholl() that calculates this is given in Box Box 7 and is called by the wrapper routine shell() that identifies the ellipsoid type and reconfigures the argument list appropriately.

As the selection of the two starting quadrants is based on a rough estimate, all 64 distances are ranked and the top three combinations taken as separate starting pairs. The routine then iterates down to the best pair of (sub-)_*n*_triangles in each (max *n* = 3) and takes the solution with the shortest separation. As a final refinement, nine points are selected around each mid-point and the closest pair taken. This solution is then checked using vec_to_egg() to find the distance from the best pair to the opposing ellipsoid. These should be identical if the true contact normal has been found. The error is typically less than 0.1%.

### 2.2 Confining children in their parent object

The **keeper** routine keeps the children of the current object inside (or on) its surface. There are only the three basic shapes to consider and the treatment of these uses much the same routines that were described in the previous section.

A parameter *keep* is set from supplied inputs for each level that sets the size of the step by which straying children are returned to their parental object. The sign of *keep* is also used as a flag to modify the confinement behaviour. If the value of *keep* is positive, then children are confined within spheres and ellipsoids but are confined to the surface of a tube and these roles are reversed when *keep* is negative. Having the default behaviour to locate children at the surface of a tube is useful both for protein secondary structures and for double-stranded nucleic acid segments. (The tube diameter is automatically set to the ideal value when these molecules are encountered).

The use of a tube for a *β*-strand is less obvious as these elements can have a marked super-helical twist. However a slightly larger tube gives space for this twist and indeed confinement on the surface encourages the chain to adopt a super-helix.

#### 2.2.1 Spheres

The packBall() routine is used by **keeper** to keep children inside a sphere and provides a simple template of the other two routines (packTube() and packEgg() described below). The code (Box 8) is self-explanatory but contains two aspects that require some elaboration.

As implemented, the code subtracts the child radius from that of the parent, so for spheres, the full body of the child will be kept inside the parent. However for elongated objects, only the radius normal to the axis of symmetry will be used so the ends of tubes and prolate ellipsoids can stick-out, whereas oblate ellipsoids will be slightly over-confined.

To save a little computation time, a margin is maintained about the surface, within which no action is taken. This is set at ±10% (margin = 0.9, margout = 1.1). Although it appears that this will save only a few arithmetic operations, it should be remembered that all geometric operations applied to objects (such as the move() utility above) are recursive and will be applied to the full underlying sub-tree of objects. The margin also prevents rapid small in/out fluctuations of children that lie close to the surface which can be visually disturbing.

#### 2.2.2 Tubes

Tubes have a cylindrical body and hemi-spherical end-caps. By default, children are constrained to lie on the surface of the cylinder and its caps. If the normal from the child centre to the tube axis intersects between the tube end-points then the child is shifted along the normal towards the surface. If outside the axial line-segment, then it is shifted in the same way as described for a sphere (above), taking the nearest end-point as a centre. If the value of the *keep* parameter is negative, then children that lie inside the tube are left unmoved. The same margin zone described above for spheres is also implemented.

An exception is made for the children of protein loop regions (which also have a tube object associated with them) but they are much less constrained and are only held inside the tube (not on its surface) with 1/10 the weight of the equivalent *α*-helix and *β*-strand. In addition, the end-cap constraints are not enforced.

An exception is also made for double-stranded nucleic acid segments where the tube enclosing the double helix is a domain level object (atomic-2). It is therefore quite undesirable to have the base-pair ‘secondary-structure’ tubes confined to this surface but rather their children (the pair of phosphates) should be on the surface. To implement this, a wrapper routine (packBase()) is used to call packTube() with a skipped generation as: packTube(grandparent,child,…) instead of the normal packTube(parent,child,…). packBase() also refines the *P*–*P* distance across the basepair and sets the axis end-points of their tube to track the phosphates. As these constraints have no chiral component, the torsion angle about the axis for basepaired phosphates is also refined, so maintaining the correct hand of the double helix.

#### 2.2.3 Ellipsoids

For ellipsoids, the **keeper** routine follows the template for the sphere but uses the utility inEgg(), which was described above, to decide who is out and who is inside. It will be recalled that inEgg() needs only the distance between the two foci of the ellipse-of-rotation to do this and so requires little computation time.

Unlike the tube end-caps, where the nearest centre was used to set the shift direction, instead, a weighted combination of the distances to both foci are used. The weights are taken as the inverse distance to each focus: so if the child lies closer to focus-1, it will have a larger weight on the component of the displacement vector in the direction of focus-1, and *vice versa*.

### 2.3 Bonds and links

The maintenance of bond and link lengths is very similar and the two routines, **bonder** and **linker**, that implement this task will be considered together. Both recursively traverse the object tree looking for things to fix.

#### 2.3.1 Bonder

*Bond lengths.* The **bonder** simply checks if an object has any assigned bonds and if so, uses the utility part2cells() to push them towards their assigned bond length.

*Nucleic acid exceptions.* Exceptions need to be made when bonding tubes in nucleic acids, which occur both at the secondary structure level as basepairs and the domain level as segments of double helix.

For basepairs, if these are part of a double helix, their ‘bond-length’ is the distance between their mid-points (object centre) which is refined to an ideal base-stacking separation. Outside a base-pair, say in a loop region, the ‘secondary structure’, like a loop in a protein, can contain multiple nucleotides and no bond length is refined.

At the domain level, double-stranded DNA segments will always be bonded end-to-end at a specific distance that allows the helix to run continuously from one segment to the next. On the other hand, when the segment is an RNA stem-loop, the chain can enter and exit the same end of the tube or, with an insertion, even through the side.

#### 2.3.2 **Linker**

*Breaking links.* The **linker** follows the same basic outline as the **bonder** but with the main difference that links can be made and broken during the simulation. The dynamic creation of links is not a built-in feature of **SimGen** and must be provided through the user-supplied **driver** routine. However, if a link becomes over stretched, it is automatically destroyed in the **linker**. The default length of a link is the bump diameter and the default extension is 50%, beyond which the link breaks.

*Preset link lengths.* Local links are automatically created for standard secondary structures, not only between the H-bonded connections in the *α*-helix, *i*–*i* + 3 and *i*–*i* + 4, but also between the *i* − 1–*i* + 1 separation along a *β*-strand. However, the non-local links between strands in a *β*-sheet must be user defined.

## 3 Examples and applications

### 3.1 Collision detection test data

#### 3.1.1 Colliding Hilbert chains

For test data, a general model of a simple macromolecule was based on a linear chain of atoms. This chain was then ‘packaged’ into a hierarchy of spherical objects, each of which contained eight children arranged as a cube. To maintain equal bond-lengths between the atoms, the path of the chain followed a recursive Hilbert curve, in which each level of the hierarchy is identical.^5^ This arrangement generates a homogeneously packed chain which allows the effects of collisions to be monitored without the added complication of variable internal structure and density.

Collisions between these objects were then engineered by applying an external displacement to propel two identical objects into each other. To avoid a direct “head-on” collision, the objects were displaced by half their radius from their line of approach. As the chain forms a cube, this means that the collision surface encompasses half a face of each cube. During the collision, the number of bumping atoms was monitored between the two objects and also within each object.

8 + 8 *crash:*

The model was initially tested with only a hard repulsion at the atomic level. However, without the protective shell of their parent, the bonds between atoms were flexible enough to allow bonded pairs to transiently pass through each other resulting in interpenetrating chains, even though these still preserved their steric and average bond lengths.

To investigate the contribution of the soft repulsion component of higher level objects, the first construct of interest involves the collision of two cubes of eight atoms. The repulsion strength at both levels was set to a value of 1 for both hard and soft modes (although the atomic level only has hard repulsion). With these values, the containing spherical shells repelled each other before the atoms could make contact.

The *soft* parameter value was then decreased, allowing inter-penetration of the high level spheres, until the internal atoms made contact. This occurred when *soft* = *hard*/5 but most of the displacement still derived from the high-level soft repulsion component and the internal arrangement of the atoms was almost unchanged. With no soft repulsion, the atomic configuration was markedly displaced and as a compromise, *soft* = *hard*/10 was taken as a combination that allowed a roughly equal contribution from each level.

64 + 64 *crash:*

**Fig. 1:**
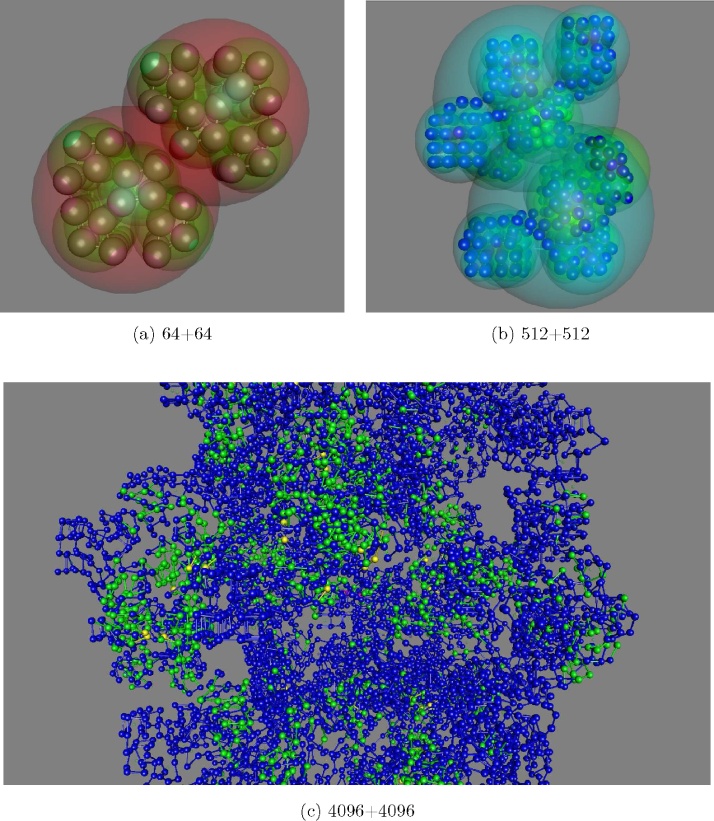
Idealised chain collisions of increasing size. (a) Two 64 atom chains are directed into each other from left and right. Groups of 8 atoms are enclosed in 8 transparent green virtual spheres which in turn are enclosed in a larger red sphere. (b) A third level is added to the hierarchy with the virtual spheres and atoms now coloured by their collision state: dark-blue designates no clash while cyan to green to yellow colours are associated with collisions of increasingly distant relatives. Cyan = same parent (cousins), green = second cousins, yellow = third. (c) The number of levels is increased to four but with smaller atoms and the virtual spheres not rendered to allow the distribution of clashes to be visualised at the atomic level. The colliding bodies remain distinct with only occasional flashes of red (fourth cousins) indicating clashes between atoms in the collision interface (which runs bottom left to top right). The distortion of the structures has been distributed evenly through many localized (green) interactions. (For interpretation of the references to colour in this figure legend, the reader is referred to the web version of this article.)

The next level of model considered was the 64 atom chain (Fig. 1(a)) and keeping the values established above for the first level in the hierarchy, an equivalent evaluation was made for the second level. As with the smaller test object, the first level spheres initially made contact when *soft* = *hard*/5, however, because of the added buffering effect of the first-level spheres, the atoms remained well separated between the two colliding objects even when *soft* = *hard*/10. To compensate for this additive contribution, the *hard* parameter value on both levels was halved and the 10% ratio to the *soft* parameter retained giving *hard* = 0.5, *soft* = 0.05 (or 50:5, as a percentage of the atomic value).

512 + 512 *crash:*

The evaluation protocol was extended to the 3-level hierarchy of 512 atoms per chain (Fig. 1(b)). Transferring the values from the previous test again led to a lack of direct contact at the atomic level and these were reduced to *hard* = 0.2, keeping the *soft* = *hard*/10 ratio. This produced a result at the mid-point of the collision (when the centroids of the bodies draw level on their collision course) that was comparable to the smaller systems.

4096 + 4096 *crash:*

For the largest model tested, with the chain packaged into 4 levels of containers, the progression of reducing the values of the *hard* and *soft* parameters was continued. However, this led to a marked number (100s) of steric violations at the atomic level both between and within the colliding bodies. Keeping the two parameters at their previous levels (*hard* = 0.2, *soft* = 0.02) the number of clashes between the bodies decreased (10s) but the number of internal violations remained high. This can be seen in Fig. 1(c) as green coloured atoms and indicates that the distortions produced by the collision are being distributed through the objects rather than concentrated at the collision interface.

As this model extends beyond the normal size range of compact biological molecules, no further experimentation was made. Of greater interest is the degree of distortion observed in the “crumple-zone” and to investigate this a more realistic protein model was used.

#### 3.1.2 Colliding multiple protein domains

**Fig. 2:**
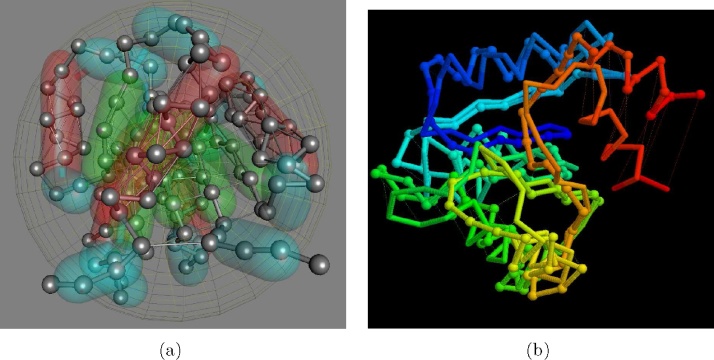
Small globular protein Che-Y used for testing. (a) The *α*-carbon backbone is drawn in a ball-and-stick representation with secondary structures contained in transparent tubes: red = *α*-helix, green = *β*-strand s and cyan = loops. Residues in these tubes are restrained to lie at the surface but only weakly for loops. Thin lines connect residues in *α* and *β* elements that are hydrogen-bonded. In some of the tests, an additional link was added between the two loop segments that connect domains (horizontal line lower-front). The ellipsoid that contains the whole domain is rendered as a feint mesh. (b) Superposition of structure before and after collision represented by a stick *α*-carbon backbone and ball-and-stick backbone, respectively. The chains are coloured from amino (blue) terminus through the spectrum to the carboxy (red) terminus. The largest deviation is seen in the C-terminal *α*-helix which contributed most to the overall 5 Åroot mean square deviation. This level was set as a target threshold to remain below. (For interpretation of the references to colour in this figure legend, the reader is referred to the web version of this article.)

Progressing to a more biologically realistic system and also to introduce a variety of container shapes, the small chemotaxis-Y protein (PDB code: 3chy), was used and modelled with tubes to contain its secondary structure elements and an ellipsoid to contain the protein. The structure was also stabilised with ‘hydrogen-bond’-like links between the *i*, …, *i* + 3 and *i*, …, *i* + 4 positions in the *α*-helices and links between hydrogen-bonded positions in the *β*-sheet (Fig. 2(a)).

A series of multi-domain models were then constructed with the protein chain as a node on a Hilbert curve giving models of 1, 8 and 64 domains. The link between domains was also optionally broken, making the domains equivalent to subunits. Collisions between these constructs were engineered as above, with the coordinates being saved at the start of the run and at the end, after the structures were well past each other. To monitor the distortion experienced by the domains/subunits during the collision, each domain in the starting structure was compared to each domain in the final structure and the smallest, mean and largest root-mean-square deviation (RMSD) recorded.

*Single domain collision:*

Two single domain structures were collided using the same parameters as determined for the equivalent sized Hilbert chains of the previous subsection (*hard* = 1.0, *soft* = 0.1). Comparing combinations of the two starting and two final structures, the mean RMS deviation was 3.6 Å, which is not far in excess of the 2.4 Åmean deviation seen when the two structures travel the same distance but do not collide. For a protein of this size, with any RMSD under 5 Å, the structures retain a clear correspondence.

Increasing the *soft* parameter to 0.2 led to less distortion (mean RMSD = 3.1) which is closer to the un-collided value. While, retaining the same *hard* : *soft* ratio with *hard* = 0.5 gave a deviation of 4.0 Å, which is still acceptable and only when the parameter values were dropped as low as *hard* = 0.5, *soft* = 0.01 was the 5 Å‘threshold’ exceed.

8 + 8 *domain collision:*

The same approach was applied to the larger 8-domain construct over a series of collisions ranging from a glancing blow to almost head-on collision. The tests were repeated both with the domains in a continuous chain and as separate (unbonded) subunits. The repulsion of the highest level sphere was set initially low with *hard* = 0.2, *soft* = 0.1 and the two *hard*/*soft* parameter combinations applied to the secondary structure and domain levels were tested. (Table 1).

The mean RMSD over the domains before and after the collision seldom exceeded the (self-imposed) threshold if 5 ÅRMSD for both parameter combinations and, as would be expected, the deviations were slightly reduced when the domains were treated as subunits. However, some of the worst distortions seen in the full (almost head-on) collisions had markedly elevated RMSD values, up to 8 Å. Despite excluding the 5 residue amino and 5 residue carboxy terminal linking segments from the comparison (which often must diverge in different directions), examination of these worst cases revealed that a large component of the error often came from displacement of the un-tethered C-terminal *α*-helix (Fig. 2(b)).

To reduce this source of error, a link as added between the mid-points of the 5-residue N- and C-terminal segments that connect domains. However, this had little effect, and even led to a slight overall increase in RMS deviations. On visual examination the distortions appeared to remain associated with the terminal helix which was still able to be markedly displaced despite the C-terminal tether but now this occurred more at the expense of disrupting other neighbouring secondary structure elements.

To allow contact at the atomic level between the colliding objects, it is desirable to limit the repulsion from the higher levels of the hierarchy. With the *hard*:*soft* parameter combinations used above of 20:10, 50:20, 50:20 for the protein, domain and secondary structure levels respectively (as a percentage of the atomic level), the colliding surfaces were able to make contact but without serious deformation occurring. These values were adopted for all further simulations.

64 + 64 *domain collision:*

The next larger complete Hilbert curve of protein domains comprises 64 domains (8256 residues) and the interaction of two such objects approaches the computational limits of what can be run on a laptop computer in real time (over a few minutes). Nevertheless, a small number of test were conducted with the highest level in the hierarchy consisting of a sphere of unlinked proteins, each composed of 8 linked domains as employed above. The *soft* : *hard* parameter combination for this level was again set to 10:5 (percent of atomic).

**Fig. 3:**
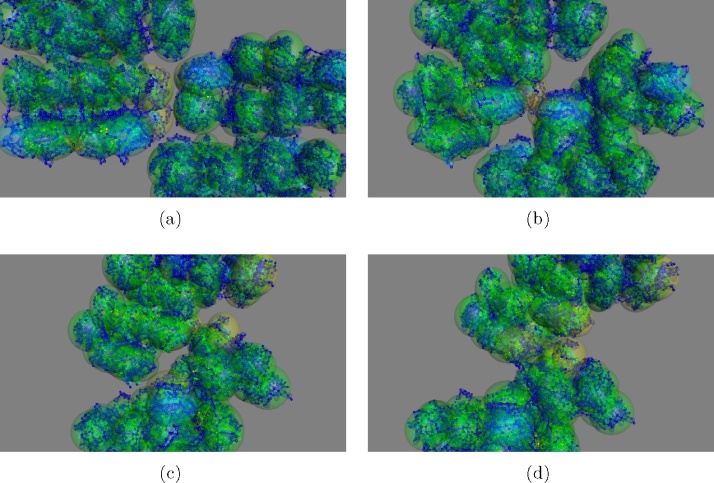
Multi-domain protein collisions. Two 8256 residue chains, arranged as 64 domains each in a Hilbert curve, are directed into each other. The frames a–d show the progression of the collision from initial contact to the point where the structures are almost past each other. Pairs of colliding objects are coloured by their separation in the structural hierarchy (as described in Fig. 1) from green for first-cousins through yellow, red and magenta for increasing levels or removal. (For interpretation of the references to colour in this figure legend, the reader is referred to the web version of this article.)

The mean domain start/final RMSD value after a half-face collision was 6.5 Å. No inter-object clashes were seen at the atomic level but a marked number of intra-object clashes built-up during the collision. It seemed likely that this higher than expected RMSD value was therefore a consequence of the speed of the collision giving insufficient time for the ‘shock-wave’ of compression to dissipate through the domains (Fig. 3).

The collision was then re-run in ‘slow-motion’ with a time-step slowed by a factor of 10. (The collision that normally took a few minutes now took 30.) The mean RMSD then dropped to the acceptable value of 5.3 Åbut intra-body clashes were still prominent during the collision. This was similar to the collision of the largest Hilbert cubes, with the intra-object collisions absorbing the ‘energy’ of the crash.

### 3.2 Distance constraint satisfaction

In this section two examples are provided using known macromolecules in which the steric exclusion (collision) parameters identified in the previous section are combined with a set of distance constraints. As the previous section used a globular protein structure, an example is taken firstly of an integral membrane protein to illustrate a different combination of objects and secondly of an RNA structure to show how the different objects can be combined to represent nucleic acid structures. Predicted distance constraints were derived from the analysis of correlated mutations (see Ref. Taylor et al., 2013 for a review).

The two examples also illustrate different strategies of constraint satisfaction. For the membrane protein, a set of protein-like starting structures is generated using a lattice based model (Taylor et al., 1994) and the predicted distance constraints are used essentially for refinement rather than to rearrange the helix packing. In this situation, the distance constraints are introduced progressively in order of their strength (probability of being correct) and only retained if they initially fall (and remain) within twice their target distance. This prevents inconsistent long range constraints disrupting the model. By contrast, the nucleic acid example has no ideal starting structure, except its (2-dimensional) secondary structure prediction and in this situation, the top 50 constraints were introduced at the start and gradually culled if they did not approach their target separation.

#### 3.2.1 Rhodopsin

The first structure of an integral trans-membrane (TM) protein to be determined was that of bacteriorhodopsin and this protein (PDB code: 1BRD) and its much larger sister family the opsins, which includes the GPCR receptors (eg: PDB code: 1GZM), remains a favourite for testing modelling and prediction methods.

**Fig. 4:**
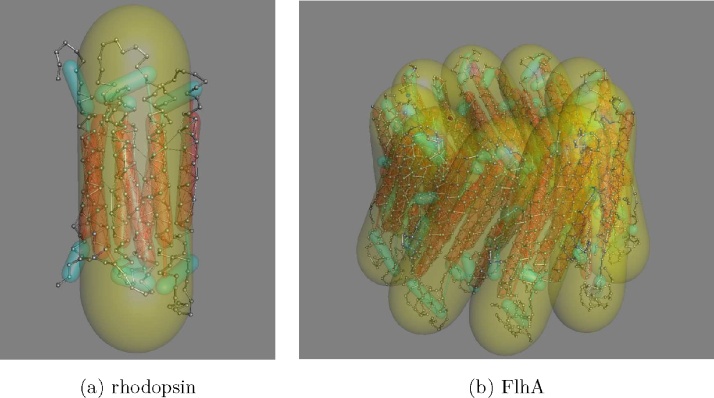
Transmembrane proteins were modelled as *α*-helix tubes inside a “kinder-surprise” confining tube (yellow), the axis of which lies perpendicular to the membrane plane. (a) A model of rhodopsin with 7-TM helices. (b) A model of the type-III secretion protein FlhA which is predicted to have 8-TM helices and is thought to form a ring of nine copies in the membrane forming a pore. (For interpretation of the references to colour in this figure legend, the reader is referred to the web version of this article.)

These structures consist of 7-TM helices arranged in a simple bundle Each was modelled as an *α*-helix confined in an tube, as described above for the small globular protein. The seven tubes were then contained in a larger tube which had a diameter narrow enough to confine the helices in a compact packing arrangement in the plane of the membrane and long enough to allow the helices to shift to a reasonable extent up and down relative to the membrane. Because the ends of the helical tubes are not constrained to lie within their containing tube, they are still free to tilt relative to each other, as is commonly observed in such structures (Fig. 4(a)).

**Fig. 5:**
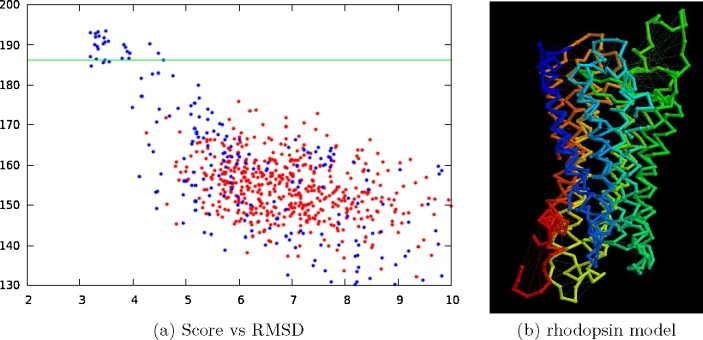
Rhodopsin predictions. (a) The RMSD of the predicted models (*X*-axis) is plotted against how well each model matches the constraints derived from the correlated mutation analysis (*Y*-axis: high is good, with the score of the native structure marked by a green line). Blue dots are from the current modelling method with red dots calculated by the FILM3 method. The RMSD is over the TM-helices only. (b) The highest scoring rhodopsin model is superposed on the native structure (PDB code 1GZM). Both structures are shown as a virtual *α*-carbon backbone coloured blue (amino) to red (carboxy) with the *α*-carbon positions rendered as small spheres on the predicted structure. The helices lie close together but deviations can be seen in the loops and at the termini. (For interpretation of the references to colour in this figure legend, the reader is referred to the web version of this article.)

As an exercise in structure refinement, the helices were allowed to move under the influence of the pairwise residue constraints derived from the correlated mutation analysis, starting from a number of configurations obtained from combinatorial enumeration over a hexagonal lattice (Taylor et al., 1994). The resulting models were then ranked on how well they had satisfied the given constraints. Plotting this score against RMSD (Fig. 5(a)), gave a clear indication for model selection and, as can be seen from the RMSD values, the highest scoring model was a good prediction (Fig. 5(b)).

The method was also applied to a protein of unknown structure, FlhA: which is a core component in the bacterial flagellum motor (in its type-III secretion sub-system) and thought to form a ring of nine proteins (Fig. 4(b)).

#### 3.2.2 SAM riboswitch

The structure of the S-adenylate-methionine type-I riboswitch (SAM-I) is a small (94 base) RNA involved in the control of bacterial gene expression (PDB code:2GIS). Consensus RNA secondary structure prediction methods (Hofacker, 2003) produce a “clover-leaf” structure reminiscent of tRNA, and like that molecule, its structure can be viewed as two basepaired hairpins (stem-loops) with each being an insertion into the other. Unlike the secondary structure prediction, the tertiary structure reveals an additional short region of base-pairing between the two hairpins (a pseudo-knot) which serves to lock the 3D structure. Interestingly, these interactions are clearly predicted by the correlation analysis and, together with the more ‘trivial’ base-pairing correlations, were used as constraints for modelling.

**Fig. 6:**
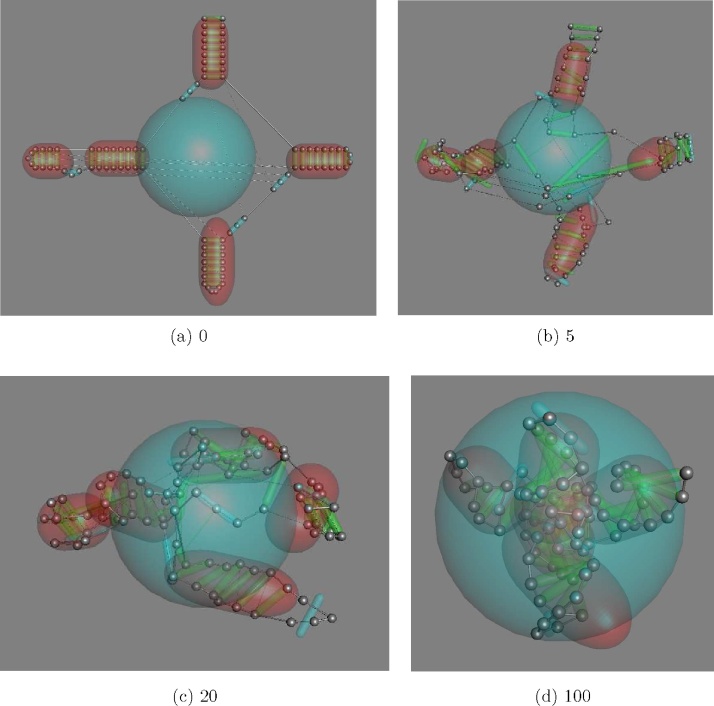
SAM riboswitch simulation in which the phosphate backbone (silver) is linked by thin green tube when basepaired (or cyan for loops) with basepaired regions (stem-loops) contained inside red tubes. The blue central sphere is the target volume inside which stem-loops aim to be contained. At the start (a, time 0), the phosphates are in their flat predicted secondary structure positions. Thin lines link pairs of phosphates with a target distance constraint with most corresponding to basepaired nucleotides. The system is simulated with random, but decreasing motion, applied to the stem-loop tubes and the structure moves rapidly to a packed conformation inside the target sphere (frames b to d). (For interpretation of the references to colour in this figure legend, the reader is referred to the web version of this article.)

The predicted base-paired regions were set-up as tubes with the phosphates of the paired bases at either end of a smaller tube forming rungs of a ladder (as described above) and these stem-loops were then specified to be confined inside a larger sphere. The flat clover-leaf secondary structure prediction was taken as a starting position for each phosphate (Fig. 6(a)) and under the influence of the confining pull (to move inside the central sphere), their bonded phosphates and the imposed distance constraints, the stem-loops moved inwards quickly (Fig. 6(b) and (c)) and packed to best accommodate the constraints (Fig. 6(d)). As not all the constraints can be simultaneously satisfied (due to prediction error), once inside the sphere, the longest constraints were gradually culled (with a stochastic bias to retain the strongest and those not within a stem-loop). By the end of a short run, a compact structure remained and, as with the TM-protein predictions, after many runs the structures were ranked on how well they satisfied the constraints.

The packing of four stem-loops has only two distinct spatial arrangements corresponding to the left and right enantiomers of a tetrahedral configuration. However, the connections between consecutive stem loops are not restricted, leading to many possible topologies (some of which are knotted). Plotting the constraint score against RMSD revealed a small but distinct bias of higher scoring models to have lower RMSDs, especially when models containing excessive *P*–*P* clashes were excluded.

However, comparing the higher scoring models against the known structure, differences were found in the orientation of the halves of stem-loops either side of the mutual “insertion” point. In the native structure the two halves of the stems are aligned but in most models the halves have a kinked alignment as there is little in the model to direct their packing away from a tetrahedral juxtaposition. While this difference accounts for most of the deviation, more importantly, even the models with the lowest RMSD had a topological differences from the native.

**Fig. 7:**
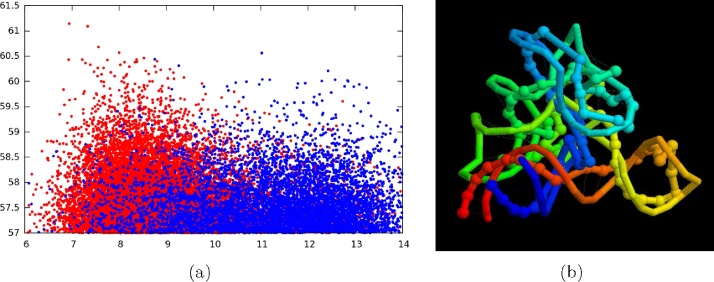
SAM riboswitch models: (a) are scored by how well they fit the top 50 constraints and this value (*Y*-axis) is plotted against the RMSD of the model from the known structure. The blue dots mark models that started from the ‘default’ secondary structure layout (Fig. 6a) and the red dots started from the alternative arrangement with two stemloops (top and right) in swapped positions. (b) The phosphate backbone of a high scoring model (ball and stick) is superposed on the known structure (stick). Both chains are coloured blue (5′) to red (3′). The cyan and yellow segments (towards the front) incorporate the long-range links that form the pseudo-knot. (For interpretation of the references to colour in this figure legend, the reader is referred to the web version of this article.)

It seemed likely that this topological error may have its roots in the restricted clover-leaf starting configuration. If all cyclic permutations of the stems around the leaf are considered, then, allowing for symmetry, there is only one other possible configuration in which two stems have been switched and this configuration also reduces the separation of the pseudo-knot distance constraints. This new starting model produced a much more distinct skew towards high-scoring, low RMSD models (Fig. 7(a)) however, the best models, despite having the correct juxtaposition of the stem-loops, still retained the same topological error (Fig. 7(b)). A more detailed analysis of this problem will be considered more fully elsewhere.

## 4 Summary and discussion

### 4.1 Summary

The behaviour of the current method has been investigated using two distinct approaches: in the examples involving collisions, a constant driving displacement was applied to a single high-level object (to force it to make contact with another object), by contrast, in the examples involving constraint satisfaction the displacements were instead applied to the lowest level objects (“atoms”). In the first case, the response of the lower level objects to maintain structural integrity was monitored whereas in the second, the resulting rearrangement of the higher levels objects was of interest.

#### 4.1.1 Collision algorithms

The algorithm described in this work for the interaction of a hierarchy of objects seeks to circumvent a fundamental problem in coarse-grained modelling which is the loss of fine detail when components become ‘bundled’ together. The “currants-in-jelly” model developed here provides a flexible approach in which the contribution of the soft high-level objects (jelly-like) can be controlled to protect the underlying atomic structure (currants) while still allowing them to interact.

Idealised macromolecular chains were used to establish the parameters to achieve this degree of interaction over a hierarchy spanning four levels. In a more realistic example using a small globular protein, the extent of the distortion experienced by the protein domain structure during collision was then examined and the parameters refined to allow an acceptable degree of deformation.

#### 4.1.2 Constraint satisfaction

The model of steric repulsion established for idealised systems was then combined with sets of predicted distance constraints, derived from correlated mutation analysis, in two differing applications. Firstly, an integral trans-membrane protein was modelled in which the packing of the seven helices was refined but without topological rearrangement. Secondly, an RNA structure was ‘folded’ under the predicted constraints, starting only from its 2-dimensional secondary structure prediction.

From the large sequence alignment available for the membrane protein, high quality distance predictions could be obtained, which combined with good starting configurations (provided by a simplified lattice model) led to the production of high-scoring models with a low RMSD to the known structure. By contrast, the RNA structure predictions had high RMSD values and although the stem-loops were correctly located in the higher-scoring models, topological differences remained which may be a result of constraints imposed by the flat starting conformation.

### 4.2 Limitations and potential

Whilst the methods and parameters established here have been shown to be effective, it is unlikely that they are optimal and many aspects of the model remain to be explored. The approach taken above was to pick reasonable values for the extent and strength of an interaction and test a few variations and combinations in the surrounding parameter-space. A more systematic approach is needed, and for this the speed of the current method is a great advantage as many simulations can be run, allowing the parameter space to be more fully explored and optimal combinations found for different types of macromolecule.

An earlier, simpler, version of the current method was used to model the dynamic interaction of actin and myosin. However, because the atomic (residue) surfaces did not interact directly in that model, artificial constraints had to be added to drive the molecular recognition events. In the current method, the residue-level surfaces can now come in contact, allowing a more realistic representation of molecular recognition based on steric compatibility. However, without a proper atomic interaction potential, the recognition of a binding event would still rely on external knowledge of the residues involved.

It was assumed that the parameters used at the start of the simulation remained the same throughout and equal for all components independently of their position or interactions. However, all these aspects could be varied and if there is a pair of objects that interact preferentially, their parameters can be set to allow their surfaces to meet whereas others might present a hard-shell repulsion. For example, in the actin/myosin example mentioned above, the actin monomers close to the myosin head could allow atomic interactions whereas all other interactions retain a high-level repulsion.

The effectiveness of the approach relies on having a rich multi-layer hierarchy of substructures. Fortunately, as seen from the varied examples provided, this is the prevalent situation for the majority of biological macromolecules. In the simulation of more homogeneous materials, such as water, it is unlikely that the approach would be useful. Similarly, for a long chain, such as DNA, although segments can be grouped like a string of sausages, the interaction of two such chains would require evaluation of the all pairs of sausages.

In large molecules, especially those composed of subunits, the components may not remain the same throughout a simulation and during an interaction, one subunit may be transferred to another assembly or a large multi-domain chain may be cleaved. In its current formulation, the program does not accommodate this, however, such events could easily be incorporated simply by updating the list of children held by each object or adding new children to the list.

### 4.3 Relationship to other methods

As outlined in the Introduction, the current method lies somewhere between a hierarchical bounding box approach (Teschner et al., 2005) and the neighbour-list approaches more commonly found in conventional molecular dynamics. Although improvements have been made since their original implementation (Verlet, 1967), such as the cell-based algorithm (Yao et al., 2004), the neighbour-list approach requires the lists to be updated frequently which involves considerable “book-keeping”, especially for objects of different shapes and sizes (Donev et al., 2005, Muth et al., 2007). By contrast in the current method, the neighbour-list is a fixed hierarchy in which the lower levels are only evaluated when higher levels collide.

Given a model in which all the constraints have been chosen well to avoid bond or bump violations at all levels, then in the absence of any user applied displacements, the behaviour of the current method is to do nothing. It is left entirely up to the user how things should move, which is done by implementing custom code in what is called the “driver” routine. This code can be either very simple, such as the few lines of code needed to implement the collision displacement or quite complicated such as the code to apply the distance constraints described in the Results section.

Extrapolating this progression, the constraints could be applied in the form of a potential and indeed a potential could even be applied to many pairs of atoms (Periole et al., 2009). The next obvious step would be to implement full molecular dynamics, Lagrangian mechanics or Monte Carlo. However, the problem with implementing anything complicated in the driver routine is that at some point, the current method will start to act on the (common) coordinates and if the driver code requires consistency in terms of distances and derived potentials, forces and velocities, then a multitude of problems will arise.

The simplest approach to this problem is to attach a warning notice stating that **SimGen** cannot be used in combination with any method that requires global internal consistency. The only route that might avoid this incompatibility would be through a more stochastic approach in which the displacements made at all levels are treated as a (semi) random Monte Carlo move of the system, which is similar to the approach of Sim et al. (2012), or perhaps exploiting the hierarchic organisation along the lines of Gipson et al. (2013). A second path to resolve the problem might be through a variation of Gaussian elastic networks (Zhang et al., 2009). However, elastic networks require a fixed topology that cannot be expected to remain intact across the large (driven) displacements envisaged for the current method.

These possible developments will be reconsidered at a later time as it is currently unclear, not only how a consistent potential could be applied but also how potentials on different levels should interact.

### 4.4 Conclusion

The method developed here provides a fast and flexible way to capture the structure of most macromolecules in a hierarchy of increasingly larger coarse-grained levels without losing the detailed low-level representation. Although much testing remains to be done, the system has the potential to be applied to very large dynamic systems including both protein and nucleic acids.

## Figures and Tables

**Table 1:** Collision induced distortions. The RMSD values observed in the small chemotaxis-Y protein (PDB code: 3chy) during the collision of two 8-domain/subunit collisions are tabulated as the minimum, average and maximum values when each domain is compared pairwise with each other before and after the collision. Four degrees of collision severity were tested from a complete **miss** through a glancing blow (**clip**) to a **half** face collision and finally an almost head-on collision (**full**). The models were tested both as individual **subunit**s and as **domains** linked in a Hilbert curve. The ‘loose’ C-terminal *α*-helix was either tethered (**link on**) or free (**no link**). Two parameter combinations were tested with the **hard:soft** repulsion ratio at both domain and secondary structure levels set to (*a*) 50:20 and (*b*) 100:20, as a percentage of the unit weight at the atomic level. The repulsion parameters for the highest protein level was held at the lower value of 20:10.

*a*	Hard:soft = 50:20											
	Subunit						Domains					
	No link			Link on			No link			Link on		
Miss	2.29	2.82	3.32	2.27	2.81	3.48	2.40	3.06	4.16	2.10	3.12	4.45
Clip	2.31	3.02	3.68	2.61	3.04	3.58	2.34	3.72	5.17	2.87	3.93	5.45
Half	2.60	3.82	5.01	2.48	3.84	5.18	2.48	3.84	5.18	3.02	4.71	8.28
Full	4.19	5.13	6.28	3.48	5.26	7.01	3.74	5.25	7.09	3.71	5.72	8.47
												

*b*	Hard:soft = 100:20											
	Subunit						Domains					
	No link			Link on			No link			Link on		
Miss	2.36	2.91	3.50	2.06	2.75	3.27	2.08	2.93	4.18	2.27	3.11	4.25
Clip	2.50	3.04	3.62	2.54	3.08	3.81	2.35	3.64	5.14	2.61	3.79	5.45
Half	2.63	3.60	4.83	2.35	3.68	4.76	2.73	4.40	7.63	2.78	4.37	6.65
Full	3.80	4.66	5.48	3.52	4.56	5.49	3.31	4.83	8.26	3.04	5.09	8.45

## Footnotes

1 In the following description the use of the terms “atom” and “atomic” are used only to indicate the lowest level in a hierarchy of objects (the leaf nodes). Although they are restricted to a spherical shape they do not necessarily represents atoms (in the physico-chemical sense).

2 Oblate and prolate ellipsoids, which have two equal axes (and hence a unique axis of symmetry) are referred to jointly as spheroids, with scalene being the remaining asymmetric type. However, to avoid confusion with spherical objects, the term will be avoided.

3 The term “speed” is used through to refer to the step size per cycle of any geometric transformation. As the stepsize is not related to any physical process, it does not have any implications for biological macromolecules.

4 The “pseudo” code in the boxed segments is actually C++ code but employs a number of macros as short-hands, most of these are obvious (like DO and FOR), however the following less obvious vector operations are used: “a|b” = the distance between points a and b, “a * b” = scalar (dot) product, “aˆb” = vector (cross) product and “a&b” = their mid-point. The Cell data structure represents an object (at any level) and encodes the number of children (kids) specified in the list: child[…]. A number of utilities operate on each object and most do what they say (e.g. moveCell(), spinCell(), part2Cells(), etc.) and apply the transform not only to the cell specified in the argument list, but recursively to all that it contains.

5 A Hilbert curve (https://en.wikipedia.org/wiki/Hilbert_curve) is the spatial equivalent of a Gray code (https://en.wikipedia.org/wiki/Gray_code) in which successive elements are only one step away from their neighbours.
